# Supplementary material for: Evidence supported by Mendelian randomization: impact on inflammatory factors in knee osteoarthritis
Source: Front Med (Lausanne). 2024 May 28;11:1382836. doi: 10.3389/fmed.2024.1382836 (PMC11165061; doi:10.3389/fmed.2024.1382836)
Supplement: Supplementary file 1 [file Data_Sheet_1.ZIP › data/GCST90274829_5e6/829.forest.pdf]

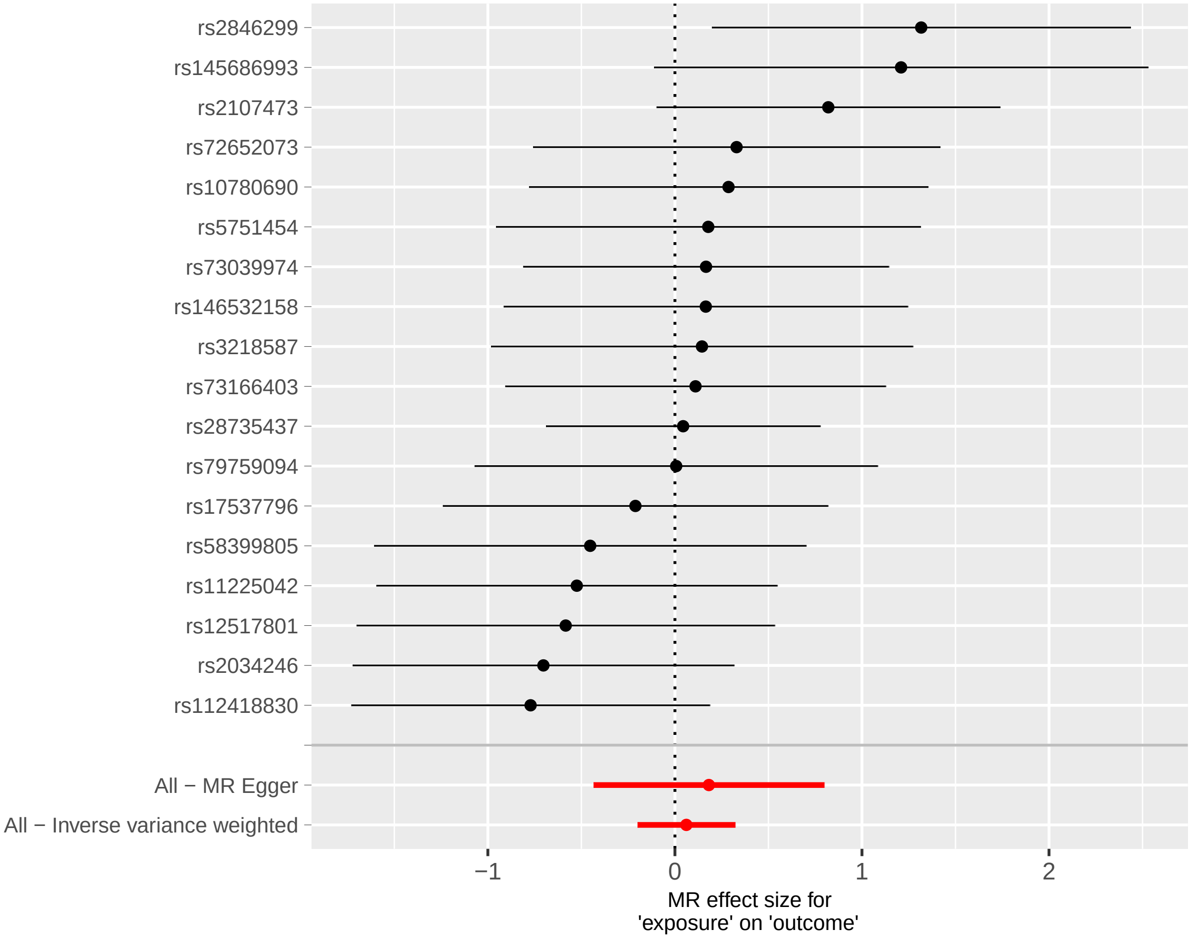
rs2846299
rs145686993
rs2107473
rs72652073
rs10780690
rs5751454
rs73039974
rs146532158
rs3218587
rs73166403
rs28735437
rs79759094
rs17537796
rs58399805
rs11225042
rs12517801
rs2034246
rs112418830
All − MR Egger
All − Inverse variance weighted
−1
0
1
2
MR effect size for
'exposure' on 'outcome'
